# Association of *MTTP* gene variants with pediatric NAFLD: A candidate-gene-based analysis of single nucleotide variations in obese children

**DOI:** 10.1371/journal.pone.0185396

**Published:** 2017-09-27

**Authors:** Dongling Dai, Feiqiu Wen, Shaoming Zhou, Zhe Su, Guosheng Liu, Mingbang Wang, Jianli Zhou, Fusheng He

**Affiliations:** 1 Shenzhen Children's Hospital, Shenzhen, China; 2 First Affiliated Hospital of Jinan University, Guangzhou, China; 3 Key Laboratory of Birth Defects, Children's Hospital of Fudan University, Shanghai, China; 4 Shenzhen Following Precision Medical Research Institute, Shenzhen, China; Universita degli Studi di Roma La Sapienza, ITALY

## Abstract

**Objective:**

We used targeted next-generation sequencing to investigate whether genetic variants of lipid metabolism-related genes are associated with increased susceptibility to nonalcoholic fatty liver disease (NAFLD) in obese children.

**Methods:**

A cohort of 100 obese children aged 6 to 18 years were divided into NAFLD and non-NAFLD groups and subjected to hepatic ultrasound, anthropometric, and biochemical analyses. We evaluated the association of genetic variants with NAFLD susceptibility by investigating the single nucleotide polymorphisms in each of 36 lipid-metabolism-related genes. The panel genes were assembled for target region sequencing. Correlations between single nucleotide variations, biochemical markers, and clinical phenotypes were analyzed.

**Results:**

97 variants in the 36 target genes per child were uncovered. Twenty-six variants in 16 genes were more prevalent in NAFLD subjects than in in-house controls. The mutation rate of *MTTP* rs2306986 and *SLC6A2* rs3743788 was significantly higher in NAFLD subjects than in non-NAFLD subjects (OR: 3.879; P = 0.004; OR: 6.667, P = 0.005). Logistic regression analysis indicated the *MTTP* variant rs2306986 was an independent risk factor for NAFLD (OR: 23.468, P = 0.044).

**Conclusions:**

The results of this study, examining a cohort of obese children, suggest that the genetic variation at *MTTP* rs2306986 was associated with higher susceptibility to NAFLD. This may contribute to the altered lipid metabolism by disruption of assembly and secretion of lipoprotein, leading to reducing fat export from the involved hepatocytes.

## Introduction

Nonalcoholic fatty liver disease (NAFLD) includes a range of liver diseases from simple fatty liver to nonalcoholic steatohepatitis (NASH), which can lead to fibrosis, cirrhosis, and hepatocellular carcinoma [1]. NAFLD is one of the most prevalent liver diseases among pediatric patients in developed countries owing to the increasing prevalence of obesity [2].

The precise pathogenesis of NAFLD remains poorly understood. Steatosis occurs when a rate of lipid influx or synthesis by hepatocytes exceeds the rate of export or catabolism [3]. The hepatic lipid metabolism pathways include hepatic *de novo* lipogenesis, lipolysis, transmembrane lipid flux, lipid oxidation, and peroxidation. An increasing number of studies identify genes that contribute to the high risk for developing pediatric NAFLD. Studies on the offspring of participants suggest a genetic predisposition to developing NAFLD [4], and heritability studies [5, 6] showed that nonalcoholic fatty liver disease is heritable. Moreover, familial aggregation studies [7] found that familial clustering of NAFLD was common. Genome-wide association studies (GWAS) of NAFLD subjects in Western countries identified several gene variants associated with NAFLD [8]. Gene expression studies reported that some genetic variants were associated with NAFLD [9].

Although insulin resistance, unhealthy diet, and sedentary lifestyle have been strongly associated with hepatic steatosis, accumulated evidence suggests that genetic background (specifically genetic polymorphisms) could be a critical factor for NAFLD predisposition in children [10, 11].

It is estimated that NAFLD affects 2.6–9.6% of pediatric patients and up to 38–53% of morbidly obese children worldwide [12]. The prevalence of NAFLD in children population is 2.1% and 68.2% among obese children in China [13]. Therefore, not every obese child develops NAFLD. We hypothesized that variants of genes in hepatic lipid metabolism pathways may contribute to increased susceptibility to pediatric NAFLD.

This study aimed to investigate the association of genetic variations with NAFLD susceptibility. We employed an approach of next-generation sequencing (NGS) and analyzed polymorphisms of 36 genes involved in hepatic lipid metabolism pathway in a cohort of children with or without NAFLD. The results of this study suggest that the genetic variation at *MTTP* rs2306986 was associated with higher susceptibility to pediatric NAFLD.

## Methods

### Study subjects

A total of 2236 children (of Han Chinese ethnicity) aged 6 to 18 years underwent regular physical examinations in 3 elementary and middle schools located in Shenzhen City, China. Among these children, 368 (16.5%) were considered obese according to the criteria adjusted with age and gender described by Cole *et al* [14].

100 of the 368 obese subjects were randomly selected and divided into a NAFLD group (group A) and a non-NAFLD group (group B). Individuals with a history of chronic liver disease (i.e., chronic hepatitis B and C, autoimmune disease, Wilson disease) as well as long-term drug consumption producing hepatic steatosis (i.e., corticosteroids), anemia, and hypothyroidism were excluded. The study protocol was approved by the Ethics Committee of Shenzhen Children's Hospital, and written informed consent was obtained from all participants' parents.

### Childhood assessments and biochemical analyses

Weight, height, waist circumference, and blood pressure of each participant were measured. The length tape measure and digital scale were accurate at 0.1 cm and 0.1 kg, respectively. BMI was calculated as body weight (kg)/height (m^2^). Adjusted BMI = (BMI of study subject)—(median of age- and gender-specific standard BMI values).

Alanine aminotransferase (ALT), aspartate aminotransferase (AST), alkaline phosphatase (ALP), total serum bilirubin (TB), direct bilirubin (DB), fasting glucose, insulin, triglyceride (TG), total cholesterol (TC), high-density lipoproteins cholesterol (HDL-C), low-density lipoproteins cholesterol (LDL-C), apolipoprotein A1 (ApoA1), and apolipoprotein B (ApoB) were determined with routine biochemical assays. Insulin resistance was evaluated through the homeostasis model assessment of insulin resistance (HOMA-IR) and calculated using the formula fasting insulin (mmol/L) × fasting glucose (mmol/L)/22.5.

### Ultrasonography and magnetic resonance imaging (MRI)

All participants underwent an ultrasonographic scan of the liver, performed by a single sonographer (Siemens Antares ultrasound machine with a CH 2- to 5-MHz convex probe). Then, a radiologist (specialized in liver imaging and blinded to the clinical and laboratory findings of the subjects) interpreted the ultrasound images. NAFLD was diagnosed using ultrasonographic scoring for liver steatosis and the findings of fatty infiltration (liver echotexture, echo penetration, and clarity of vessel structures) [15].

Subjects with the suggested NAFLD by ultrasonography were confirmed by MR imaging with a standard torso phased-array coil centered over the liver at 3-T MR imager (Signa Excite HD; GE Medical Systems, Milwaukee, WI; eight-channel coil). Two experienced radiologists reviewed images through Osirix and estimated the liver proton density fat fraction (PDFF), which is a measure of liver fat content [16].

### Targeted capture and next-generation sequencing

Genomic DNA was extracted from 2 ml of ethylenediaminetetraacetic acid (EDTA) anticoagulated peripheral blood using a Qiagen DNA isolation kit (Qiagen, Valencia, CA), fragmented and used for sample library construction (Illumina Hiseq) according to the manufacturer's instructions.

Briefly, 1 μg of genomic DNA in 100μl of TE was fragmented to a pool 150–250 bp by Bioruptor (Diagenode, Belgium), and then adapters (Invitrogen, USA) were ligated to both ends of the resultant fragments. The adapter-ligated templates were purified by the MagPure A3 XP beads (Magen, China). The purified DNA was amplified by ligation-mediated polymerase chain reaction, purified, and hybridized to lipid metabolism-related genes (LMRG) panel (iGeneTech, China) for enrichment. The target genes in LMRP panel including: *TM6SF2*, *ACSS1*, *GCKR*, *ACSS3*, *ACACB*, *NR1I2*, *SREBF1*, *SREBF2*, *DGAT2*, *DGAT1*, *TNF*, *LPL*, *FASN*, *APOB*, *NCAN*, *FDFT1*, *PEMT*, *FATP2 (SLC27A2)*, *DLAT*, *SLC6A2*, *MTTP*, *PPP1R3B*, *ADIPOQ*, *CYP2E1*, *PPARG*, *LEP*, *CPT1*, *UCP3*, *UCP1*, *PPARA*, *LIPE (HSL)*, *SLC25A20 (CACT)*, *LIPC (HL)*, *PNPLA3*, *PNPLA2 (ATGL)*, *CPT2*. The hybridized fragments were bound to Streptavidin Dynabeads (Invitrogen, USA) and washed with proper stringent buffers (iGeneTech, China). The captured products were quantified with the Qubit dsDNA HS Assay Kit (Invitrogen, USA). Paired-end sequencing, which reads 150 bases from each end of the fragment for targeted libraries, was performed using Illumina HiSeq Xten and Illumina MiSeq instrumentation (Illumina, San Diego, CA).

### Genetic variation detection and verification

Generated sequences in the clean reads were mapped the NCBI human reference genome (hg19/GRCh37) with Burrows-Wheeler Aligner, after using a quality filter (Trimmomatic) to remove reads containing sequencing adapters and low-quality reads. A low-quality read was defined as quality score less than 20 or a read shorter than 40 bases. Duplicates were marked using Picard (v1.54) software (http://picard.sourceforge.net/). GATK (Genome Analysis Toolkit) was used for calling SNPs and InDels. Annotation and classification for SNPs and InDels were obtained through ANNOVAR. The data was identified by dbSNP database (http://www.ncbi.nlm.nih.gov/projects/SNP/snp_summary.cgi), 1000 human genomes database (www.1000genomes.org/), and iGeneTech database (a database that is built on Whole Exome Sequencing based study of genetic risk for NAFLD, consisting of 2000 healthy Chinese people across China). Among the iGeneTech database, the 800 Han Chinese subjects were used as in-house controls. The inhouse controls were confirmed without NAFLD, metabolic disorders, diabetes mellitus, obesity, autoimmune hepatitis, dyslipidemia, or any family history of above diseases.

The variants were then selected using additional filter as following steps. First, the mutations in untranslated regions and splicing sites were removed. Then, the variants without functional prediction in at least one of the 4 algorithms (SIFT23, PolyPhen-2, Mutation Taster, and GERP++) that we used to investigate disease-causing potentials were discarded. Furthermore, the alterations that had more than 15% minor allele frequency (MAF) in one of the three databases of 1000 genomes, ESP6500si, and iGeneTech, or without MAF reported in the three databases were filtered. Finally, mutations without identification were excluded. The selected mutations were verified by Sanger sequencing.

### Statistical analysis

SPSS v19.0 statistical software (StataCorp) was used for all the statistical analyses. Continuous variables were represented as the means ± SD. The two-tailed *t*-test was used for comparison of continuous variables across groups, while the Chi-squared (χ^2^) test and 1-factor ANOVA were used for comparisons of categorical variables. A P-value <0.05 was considered statistically significant. Potential associations between each single nucleotide variations (SNV) and NAFLD were tested using a χ^2^ test for single SNP associations. The pair of the two SNVs was entered as a logistic regression model using Enter selection, and adjusted for the appropriate demographic variables and metabolic covariates.

## Results and discussion

### Subject characteristics

Thirty-nine (39%) of the 100 randomly selected obese participants were diagnosed with NAFLD. Age, sex, height, and systolic blood pressure (SBP) were not significantly different between the two groups (each P > 0.05). However, compared to the non-NAFLD group, NAFLD group subjects had higher waist circumference (WC), weight, BMI, and adjusted BMI values as well as higher levels of ALT, ALP, TG, TC, FFA, LDL-C, and ApoB (P < 0.05 for all parameters). However, there was no significant difference between the two groups in the levels of glucose, insulin, HOMA-IR, AST, TB, DB, HDL-C, and ApoA1 (P > 0.05 for all parameters). The demographic and biochemical characteristics of the study groups are described in Table 1.

**Table 1:** Anthropometric and biochemical characters in NAFLD and non-NAFLD groups (of Han Chinese ethnicity).

Variables	NAFLD (N = 39)	Non-NAFLD (N = 61)	T	P value
**Sex, M/F**	19/20	37/24	1.376	0.241
**Age at diagnosis (years)**	13.41 ± 2.26	13.54 ± 2.41	0.271	0.787
**Systolic BP (mmHg)**	123.15 ± 17.62	120.21 ± 12.00	-0.981	0.329
**Height (cm)**	162.79 ± 12.61	162.07 ± 13.29	-0.266	0.791
**Weight (kg)**	78.78 ± 16.84	68.27 ± 13.94	-3.225	0.002
**BMI (kg/m2)**	29.64 ± 3.76	26.19 ± 2.63	-5.343	<0.001
**Adjusted BMI (kg/m2)**	4.66 ± 0.59	0.71 ± 0.39	-5.794	<0.001
**WC (cm)**	98.07 ± 9.31	87.41 ± 8.37	-5.380	<0.001
**TC (3.1–5.8 mmol/L)**	4.58 ± 0.98	3.61 ± 0.71	-5.715	<0.001
**TG (0.23–1.7 mmol/L)**	1.75 ± 0.74	1.08 ± 0.47	-5.531	<0.001
**FFA (2.07–4.1 mg/dL)**	0.63 ± 0.20	0.53 ± 0.17	-2.558	0.013
**HDL-C (0.9–1.8 mmol/L)**	1.08 ± 0.28	1.06 ± 0.19	-0.151	0.709
**LDL-C (2.07–4.1 mmol/L)**	3.03 ± 0.68	2.45 ± 0.49	-4.617	<0.001
**ApoA1 (1.05–2.05 g/L)**	1.23 ± 0.25	1.19 ± 0.23	-0.744	0.459
**ApoB (0.55–1.3 g/L)**	1.04 ± 0.18	0.85 ± 0.24	-4.549	<0.001
**ApoB/ApoA1**	0.786 ± 0.037	0.779 ± 0.038	-0.118	0.906
**Glucose (3.1–5.6 mg/dL)**	5.42 ± 0.43	4.92 ± 0.15	-1.104	0.275
**Insulin (1.9–23 μU/mL)**	20.94 ± 2.90	31.65 ± 7.55	0.602	0.549
**HOMA-IR**	4.79 ± 0.72	3.43 ± 0.68	-1.364	0.176
**TB (0.9–17.1 μmol/L)**	10.54 ± 0.84	9.57 ± 0.53	-0.969	0.336
**DB (0–6.08 μmol/L)**	3.07 ± 0.48	2.38 ± 0.147	-1.623	0.108
**ALT (0–40 IU/L)**	65.44 ± 10.83	17.93 ± 1.05	-5.439	<0.001
**AST (0–40 IU/L)**	40.77 ± 4.79	32.44 ± 6.31	-1.562	0.120
**ALP (40–500 IU/L)**	246.90 ± 13.56	264.92 ± 7.85	2.921	0.005
**Lipid content**	21.60 ± 3.19	11.25 ± 1.63	-3.169	0.002

WC, waist circumference; ALT, alanine aminotransferase; AST, aspartate aminotransferase; ALP, alkaline phosphatase; TB, total serum bilirubin; DB, direct bilirubin; TG, triglyceride; TC, total cholesterol; HDL-C, high-density lipoproteins cholesterol; LDL-C, low-density lipoproteins cholesterol; ApoA1, apolipoprotein A1; ApoB, apolipoprotein B.

### Mutational analysis of genes

The variants that were not on target were excluded, resulting in 494 variants within the 36 target genes per subject (S1 Table). After completion of analysis steps by the functional filter described in Methods, 97 nonsynonymous exonic variants per patient were verified (S2 Table). All the mutations were scored as 'damaging' by at least 1 of the 4 algorithms (SIFT23, PolyPhen-2, Mutation Taster and GERP++). Mutation rates in the NAFLD subjects, non-NAFLD subjects and the in-house controls were compared, using Fisher's Exact Test (S3 Table). Twenty-six SNVs were found to be enriched in the subjects with NAFLD when compared with in-house controls (all P < 0.05) (S3 Table). The 26 SNPs were located in 16 genes; *MTTP* rs2306986 and *SLC6A2* rs3743788 were significantly higher in subjects with NAFLD compared to non-NAFLD (OR: 3.879; P = 0.004; OR: 6.667, P = 0.005, respectively), see Table 2.

**Table 2:** Comparison of mutation rate in *MTTP* rs2306986 and SLC6A2 rs3743788 between NAFLD group and non-NAFLD group (of Chinese Han ethnicity).

SNV	NAFLD (n = 39)	Non-NAFLD (n = 61)	χ^2^ value	OR	P-value
*MTTP* rs2306986	19/20	12/49	9.331	3.879	0.004
*SLC6A2* rs3743788	10/29	3/58	7.294	6.667	0.005

We further compared physical and biochemical findings between the subjects with and without variants of the two genes, and found that WC and the levels of ALT, TC, LDL, lipid content, and ApoB were significantly higher in the subjects with *MTTP* rs2306986 variant (P = 0.025, 0.001, 0.001, 0.005, 0.002, and <0.001, respectively), as shown in Table 3. The level of TG, TC, and ApoB was significantly higher in the subjects with *SLC6A2 rs3743788* variant (p = 0.007, 0.029, and 0.003, respectively), as shown in Table 4. Binary logistic regression analysis indicated the *MTTP* rs2306986 was a risk factor for NAFLD (OR: 3666.537, P = 0.043), as shown in Table 5.

**Table 3:** The comparison of anthropometric and biochemical characteristics based on the presence of variation of *MTTP* rs2306986 (subjects of Han Chinese ethnicity).

Variables	*MTTP* rs2306986		T-test or χ^2^ test	
	A (N = 69)	B (N = 31)	T or F	P value
**Sex (M/F)**	25/44	9/22	0.494	0.482
**Age**	13.23 ± 2.55	14.06 ± 1.67	1.662	0.100
**Height**	160.98 ± 13.91	165.33 ± 10.21	1.530	0.129
**Weight**	71.15 ± 16.63	75.36 ± 14.24	1.216	0.227
**BMI**	27.38 ± 3.63	27.86 ± 3.31	0.625	0.534
**Adjusted BMI**	1.844 ± 0.46	3.15 ± 0.66	1.588	0.116
**WC**	90.04 ± 10.63	95.38 ± 8.02	2.284	0.025
**SBP**	120.58 ± 14.06	123.13 ± 15.52	0.807	0.422
**Glucose**	5.14 ± 0.28	5.09 ± 0.17	-0.111	0.912
**Insulin**	31.96 ± 15.49	17.51 ± 2.81	-0.626	0.533
**HOMA-IR**	4.02 ± 0.68	3.85 ± 0.59	-0.151	0.881
**TB**	9.90 ± 0.54	10.05 ± 0.87	0.145	0.885
**DB**	2.38 ± 0.15	3.25 ± 0.58	1.958	0.053
**ALT**	25.48 ± 2.30	60.90 ±13.91	3.585	0.001
**AST**	24.35 ± 9.91	49.90 ± 18.78	2.023	0.046
**ALP**	277.90 ± 80.95	272.39 ± 81.19	-0.315	0.754
**TG**	1.32 ± 0.08	1.38 ± 0.12	0.435	0.664
**Cholesterol**	3.77 ± 0.87	4.46 ± 0.94	3.593	0.001
**FFA**	0.56 ± 0.02	0.58 ± 0.03	0.277	0.783
**HDL**	1.05 ± 0.03	1.12 ± 0.05	1.533	0.129
**LDL**	2.55 ± 0.60	2.93 ± 0.62	2.894	0.005
**ApoA1**	1.18 ± 0.24	1.25 ± 0.23	1.175	0.243
**ApoB**	0.84 ± 0.03	1.11 ± 0.02	6.044	0.000
**ApoB/ApoA1**	0.778 ± 0.035	0.792 ± 0.041	-0.257	0.798
**Lipid content**	12.82 ± 2.00	20.77 ± 2.80	-0.125	0.026
**Diagnosis (N/n)**	20/49	19/12	10.148	0.002

A, without variation; B, with variation; N, NAFLD; n, non-NAFLD; WC, waist circumference; ALT, alanine aminotransferase; AST, aspartate aminotransferase; ALP, alkaline phosphatase; TB, total serum bilirubin; DB, direct bilirubin; TG, triglyceride; TC, total cholesterol; HDL-C, high-density lipoproteins cholesterol; LDL-C, low-density lipoproteins cholesterol; ApoA1, apolipoprotein A1; ApoB, apolipoprotein B.

**Table 4:** The comparison of anthropometric and biochemical characters between subjects with and without the *SLC6A2* rs3743788 variant (subjects of Han Chinese ethnicity).

Variables	*SLC6A2* rs3743788		T-test or χ^2^ test	
	A (N = 87)	B (N = 13)	T or F	P value
**Sex (M/F)**	56/31	10/3	0.333	0.564
**Age**	13.43 ± 2.42	13.84 ± 1.67	-0.587	0.559
**Height**	161.58 ± 13.41	167.26 ± 8.46	-1.479	0.143
**Weight**	71.36 ± 16.08	79.84 ± 13.43	-2.062	0.054
**BMI**	27.41 ± 3.61	28.26 ± 2.94	-0.983	0.360
**Adjusted BMI**	2.08 ± 0.41	3.33 ± 1.05	-1.106	0.285
**WC**	91.19 ± 10.21	94.64 ± 9.71	-1.132	0275
**SBP**	121.19 ± 13.63	122.69 ± 19.92	-0.263	0.797
**Glucose**	5.13 ± 0.22	5.04 ± 0.33	0.224	0.825
**Insulin**	28.33 ± 12.26	21.47 ± 6.06	0.502	0.617
**HOMA-IR**	3.77 ± 0.52	5.23 ± 1.80	-0.777	0.450
**TP**	71.09 ± 4.14	70.38 ± 3.99	0.600	0.557
**ALB**	42.21 ± 3.48	41.84 ± 2.87	0.433	0.670
**TB**	10.12 ± 0.58	8.78 ± 1.00	1.197	0.246
**DB**	2.70 ± 0.23	2.32 ± 0.43	0.780	0.445
**ALT**	33.06 ± 4.83	59.23 ± 17.71	-1.426	0.176
**AST**	31.98 ± 6.78	34.23 ± 5.39	-0.260	0.796
**ALP**	279.32 ± 82.05	255.23 ± 69.74	1.134	0.272
**TG**	1.27 ± 0.07	1.80 ± 0.18	-2.793	0.013
**TC**	3.91 ± 0.92	4.52 ± 0.98	-2.108	0.052
**FFA**	0.57 ± 0.02	0.56 ± 0.05	0.083	0.935
**HDL-C**	1.08 ± 0.02	1.04 ± 0.05	0.514	0608
**LDL-C**	2.64 ± 0.63	2.89 ± 0.62	-1.354	0.195
**ApoA1**	1.21 ± 0.23	1.20 ± 0.26	0.119	0.906
**ApoB**	0.89 ± 0.22	1.10 ± 0.26	-2.747	0.015
**ApoB/ApoA1**	0.787 ± 0.029	0.751 ± 0.069	0.477	0.640
**Lipid content**	15.19 ± 1.85	15.89 ± 3.33	-0.180	0.859
**Diagnosis (N/n)**	29/58	10/3	9.033	0.003

A, without variation; B, with variation; N, NAFLD; n, non-NAFLD; WC, waist circumference; ALT, alanine aminotransferase; AST, aspartate aminotransferase; ALP, alkaline phosphatase; TB, total serum bilirubin; DB, direct bilirubin; TG, triglyceride; TC, total cholesterol; HDL-C, high-density lipoproteins cholesterol; LDL-C, low-density lipoproteins cholesterol; ApoA1, apolipoprotein A1; ApoB, apolipoprotein B.

**Table 5:** Logistic regression for the two significant variants in subjects with and without NAFLD (subjects of Han Chinese ethnicity).

Model term	B	S.E.	Wald	OR	95% C.I.	P value
Constant	-137.113	62.971	4.741	0.000		0.029
Ajusted MBI	1.822	0.881	4.275	6.185	1.099, 34.794	0.039
WC	0.749	0.350	4.588	2.115	1.066, 4.196	0.032
HOMAIR	0.321	0.230	1.952	1.378	0.879, 2.162	0.162
TG	4.300	2.602	2.732	73.736	0.450, 12090.635	0.098
FFA	26.608	13.050	4.157	3.595E11	2.802, 4.613E22	0.041
TC	4.578	2.430	3.551	97.335	0.832, 11383.280	0.060
LDL-C	2.151	4.610	0.218	8.593	0.001, 72201.079	0.641
ApoB	14.032	8.067	3.026	1241762.292	0.169, 9.130E12	0.082
*MTTP* rs2306986	8.207	5.605	2.144	3666.537	0.062, 2.165E8	0.043
SLC6A2 rs3743788	0.608	2.298	0.070	1.837	0.020, 165.875	0.791
SLC6A2 rs3743788* *MTTP* rs2306986	-4.628	4.862	0.906	0.010	0.000, 134.467	0.341

BMI, body mass index; HOMA-IR,homeostasis model assessment of insulin resistance; WC, waist circumference

## Discussion

This study revealed several interesting findings in phenotypes and genotypes of children with NAFLD.

NAFLD was detected in 39% of the obese children in this study—lower than the 68.7% reported by Kodhelaj *et al* [17], 55.1% by Lin *et al* [18], and 42.9% by Duarte *et al* [19], but higher than the percentages reported by Pozzato *et al* (34.6%) [20] and Guijarro *et al* (30%) [21]. The difference in NAFLD may reflect the differences among the ethnic populations.

We found that the BMI was significantly higher in the NAFLD group than in the non-NAFLD group, thus demonstrating that BMI may have significantly contributed to pediatric NAFLD development. This finding was consistent with the report that BMI was an independent risk factor for the formation of fatty liver [22].

On the other hand, we found no significant difference between the two groups in levels of insulin, glucose, and HOMA-IR. As previously reported, NAFLD was not associated with insulin secretion and insulin sensitivity in young obese children with strictly matched sex, age, pubertal status, and BMI [23]. These findings further supported our focus on hepatic lipid metabolism in this study [24].

Selecting candidate genes is challenging in the study of genetic polymorphism of NAFLD. To avoid arbitrariness, we selected the 36 genes involved in hepatic lipid metabolism in various ways including lipid synthesis, transmembrane lipid transport, lipolysis, and lipid oxidation. 494 variants in the 36 genes per subject were detected in this cohort, and 97 of them were identified in each patient after functional filtration. Twenty-six variants in 16 genes were more prevalent in NAFLD subjects than in-house controls, but did not differ from non-NAFLD subjects.

Among the 16 genes, *ACACB*, *SREBF1*, *FASN*, *ACSS3*, and *DGAT2* involve lipid synthesis; *APOB*, *SLC27A2*, *MTTP*, *TNF*, and *SREBF1* participate in lipid influx and export from liver cells; *LIPE* (*HSL*), *PNPLA2* (*ATGL*), and *PNPLA3* are involved in lipolysis; and *ACACB*, *DLAT*, *PPARG*, *ADIPOQ*, and *CPT2* are involved in lipid oxidation. Thus, 16 genes could be associated with obesity in children. For instance, several of them have been identified in previous obesity studies including *ACACB*, *ACSS*, *ADIPOQ*, *DGAT2*, *HSL*, *FASN*, *PNPLA2*, *PNPLA3*, *PPAR-γ*, *SREBP1* SNP17 and *TNF* [25–37].

Furthermore, we found that the mutation rate of *MTTP* rs2306986 (c.294G>C, p.E98D) and *SLC6A2* rs3743788 (c.1646T>C,p.I549T) was significantly higher in subjects with NAFLD than that without NAFLD. Our results suggested that the two SNVs were associated with NAFLD in obese children. Triglycerides are either incorporated into VLDL particles for export or stored within the hepatocyte. Variations in lipid metabolism may lead to different rates of lipid accumulation in the hepatocyte.

The human microsomal triglyceride transfer protein (*MTTP* or *MTP*) carries lipid transfer function and is critical for the assembly and secretion of very-low-density lipoprotein (VLDL) to remove lipid from liver. Thus, changes in the liver lipid secretion efficiency (mediated by *MTTP*) can lead to hepatic steatosis [38]. Several lines of evidence have shown that *MTTP* polymorphisms may modulate the lipid homeostasis and may eventually lead to a high risk for NAFLD if such function is compromised because of genetic variation.

A large number of genetic polymorphisms in the *MTP* gene have been identified. In *MTTP*-knockout mice, there was a striking reduction in VLDL triglyceride accompanied by hepatic steatosis [39, 40]. The *MTP* -493G/T and GG polymorphism (rs1800591) have been implicated in the pathogenesis of NAFLD [41–44]. The GG genotype was associated with increased steatosis and histological NASH grade in NASH patients [45–48]. The 297H (rs2306985) variant increased the NAFLD risk by interaction with age, insulin resistance, and BMI [49]. The SNP -164 T/C (rs1800804) was associated with an increased risk of NAFLD in the Han Chinese population according to Peng *et al* [50].

These studies reasoned that common functional polymorphism in the human *MTP* gene may result in decreased protein production and inefficient regulation of hepatic lipid metabolism, thus contributing to the development of NAFLD [38, 51]. The mutation identified at rs2306986 in this study represents a new *MTP* variant and the impact on the function, as was predicted by PolyPhen-2, ranked as “possible damaging” with a score of 0.712 (sensitivity: 0.86; specificity: 0.92). This variant may alter gene expression to impair the function of MTP protein, contributing to the development of NAFLD.

Possible involvement of *SLC6A2* in NAFLD pathogenesis has not been investigated. *SLC6A2* gene encodes the norepinephrine transporter (NET), which is responsible for reuptake of norepinephrine into presynaptic nerve terminals and is a regulator of norepinephrine homeostasis. NET exerts a fine regulation of norepinephrine-mediated behavioral and physiological effects including mood, depression, feeding behavior, and cognition [52]. Individual variations in this gene were implicated in susceptibility to abnormal human behavior including depression and attention deficit [53]. Different combinations of T-182C and the G1287A polymorphisms of NET gene might increase morbidity risk in major depressive subpopulations [54]. In patients with major depressive disorder, there seemed to be a relationship between the volume of the dorsolateral prefrontal cortex and polymorphism of the *SLC6A2* G1287A gene [54]. Furthermore, there was a correlation between the NET T1-82C polymorphism and the susceptibility to depression [55–57].

Depression was reported to be a risk factor for NAFLD [58]. The major depressive disorder was associated with more severe liver steatosis and poor treatment outcomes in patients with NAFLD [59]. In patients with NAFLD, depression was associated with more severe ballooning changes in hepatocytes [60]. Childhood obesity was associated with depression as reported by an Australia study [61]. Taken together, *SLC6A2* polymorphisms may indirectly impact hepatic lipid metabolism by swinging psychological mood in obese children.

Moreover, the Reactome study (www.reactome.org) indicated that SLC6A2 (NET1) was associated with transport of hexose (glucose, fructose, metal ions), which correlated with coronary artery disease, height, glucose, and blood pressure according to the genome-wide association study. Furthermore, reactome reports that norepinephrine and epinephrine inhibit insulin secretion and they are the substrate of NET1; NET1 function is inversely regulated by insulin [62]. NAFLD is closely associated with insulin resistance and type 2 diabetes. The association of *SLC6A2* polymorphisms with NAFLD may be mediated through insulin resistance.

There are limitations in this study. First, this cohort consisted of a relatively small sample and therefore our results need to be verified in multicenter-based large cohorts. Second, genetic variants detected in NAFLD should also be compared with well-matched normal healthy subjects, not just with in-house controls. Third, *MTP* appeared to be an important gene and its variants may have altered lipid metabolism, leading to NAFLD in obese children. However, we were not able to analyze MTP expression at mRNA and protein levels in this cohort. Finally, the ethnicity limitation was that only Han Chinese subjects were included in the present study and the genetic risk factor for NAFLD may differ among different ethnicities.

## Conclusions

In this study, we analyzed genetic variants of 36 genes involved in lipid metabolism in 100 obese children. We found that the *MTTP* rs2306986 (p < 0.05) and *SLC6A2* rs3743788 (p < 0.05) variants were significantly associated with NAFLD. The presence of SNV (rs2306986) in the *MTTP* gene was an independent risk factor for the susceptibility to NAFLD in obese children while the *SLC6A2* polymorphism may exert indirect effect on the development of NAFLD. The identified association of gene polymorphism and NAFLD may point to a more effective treatment strategy.

## Supporting information

S1 Table The 494 variants were detected among the 36 target genes per subject (of Han Chinese ethnicity) after using a quality filter (Trimmomatic) to remove reads containing sequencing adapters and low-quality reads. (XLS)

S2 Table Nonsynonymous SNVs in target region sequencing (subjects of Han Chinese ethnicity). A total of 97 nonsynonymous exonic variants per patient were verified within the 36 target genes per subject. All the mutations were scored as 'damaging' by at least 1 of the 4 algorithms (SIFT23, PolyPhen-2, Mutation Taster and GERP++).
(DOC)

S3 Table The distributions of 26 SNVs and the comparison between NAFLD, non-ANFLD groups and in-house controls (of Han Chinese ethnicity). The mutations were compared using Fisher's exact test. The 26 SNVs located in 16 genes were enriched in the NAFLD subjects compared to in-house controls (all P < 0.05).
(DOC)
