# Supplementary material for: Association of MTTP gene variants with pediatric NAFLD: A candidate-gene-based analysis of single nucleotide variations in obese children
Source: PLoS One. 2017 Sep 27;12(9):e0185396. doi: 10.1371/journal.pone.0185396 (PMC5617203; doi:10.1371/journal.pone.0185396)
Supplement: S2 Table — A total of 97 nonsynonymous exonic variants per patient were verified within the 36 target genes per subject. All the mutations were scored as 'damaging' by at least 1 of the 4 algorithms (SIFT23, PolyPhen-2, Mutation Taster and GERP++). (DOC) [file pone.0185396.s002.doc]

**S2 Table. 97 nonsynonymous SNVs in target region sequencing (subjects of Han Chinese ethnicity)**

| Chr | snp | BP (hg18) | cytoBand | allele | function | Fisher.P  NAFLD vs non-NAFLD | MaxFreq | NAFLD | Non-NAFLD | ALL | 1000g2012 | ESP6500 | iGeneTechBase | Gene |
| --- | --- | --- | --- | --- | --- | --- | --- | --- | --- | --- | --- | --- | --- | --- |
| chr1 | rs2229291 | 53676401 | 1p32.3 | T/G | nonsynonymous | 0.688481682 | 0.0935419 | 12 | 24 | 36 | 0.06 | 0.007151 | 0.0935419 | CPT2 |
| chr1 | rs142600166 | 53676865 | 1p32.3 | G/A | nonsynonymous | 0.396039604 | 0.000538 | 1 | 0 | 1 | 0.0005 | 0.000538 | - | CPT2 |
| chr2 | rs1801702 | 21225485 | 2p24.1 | C/G | nonsynonymous | 1 | 0.06 | 1 | 2 | 3 | 0.06 | 0.049208 | 0.0176431 | APOB |
| chr2 | rs183117027 | 21227212 | 2p24.1 | C/T | nonsynonymous | 0.523005242 | 0.0036618 | 0 | 2 | 2 | 0.0014 | - | 0.0036618 | APOB |
| chr2 | rs2163204 | 21231387 | 2p24.1 | T/G | nonsynonymous | 0.707548204 | 0.0203063 | 2 | 6 | 8 | 0.02 | 0.000923 | 0.0203063 | APOB |
| chr2 | rs200143030 | 21232409 | 2p24.1 | C/T | nonsynonymous | 1 | 0.0005 | 0 | 1 | 1 | 0.0005 | 0.000154 | - | APOB |
| chr2 | rs184512808 | 21233189 | 2p24.1 | T/C | nonsynonymous | 0.396039604 | 0.0013316 | 1 | 0 | 1 | 0.0005 | - | 0.0013316 | APOB |
| chr2 | rs13306187 | 21236085 | 2p24.1 | C/T | nonsynonymous | 0.300456099 | 0.0066578 | 3 | 1 | 4 | 0.0018 | 0.000384 | 0.0066578 | APOB |
| chr2 | rs367788462 | 21251402 | 2p24.1 | C/A | nonsynonymous | 1 | 0.0006658 | 0 | 1 | 1 | - | - | 0.0006658 | APOB |
| chr2 | rs13306194 | 21252534 | 2p24.1 | G/A | nonsynonymous | 0.626023834 | 0.0439414 | 9 | 11 | 20 | 0.04 | 0.000231 | 0.0439414 | APOB |
| chr2 | rs13306198 | 21260084 | 2p24.1 | G/A | nonsynonymous | 0.539428643 | 0.0219707 | 6 | 6 | 12 | 0.01 | 0.000384 | 0.0219707 | APOB |
| chr2 | rs186544754 | 21263905 | 2p24.1 | C/A | nonsynonymous | 1 | 0.0016644 | 3 | 4 | 7 | 0.0014 | - | 0.0016644 | APOB |
| chr2 | rs200464882 | 21266399 | 2p24.1 | C/G | nonsynonymous | 1 | 0.0005 | 0 | 1 | 1 | 0.0005 | - | - | APOB |
| chr3 | rs371659081 | 119530434 | 3q13.33 | G/A | nonsynonymous | 1 | 0.000154 | 0 | 1 | 1 | - | 0.000154 | - | NR1I2 |
| chr3 | rs370394566 | 119536042 | 3q13.33 | G/A | nonsynonymous | 1 | 0.0003329 | 0 | 1 | 1 | - | 0.000077 | 0.0003329 | NR1I2 |
| chr3 | rs1801282 | 12393125 | 3p25.2 | C/G | nonsynonymous | 0.150479129 | 0.090574 | 1 | 8 | 9 | 0.07 | 0.090574 | 0.0189747 | PPARG |
| chr3 | rs138773406 | 186572419 | 3q27.3 | C/A | nonsynonymous | 0.396039604 | 0.0018 | 1 | 0 | 1 | 0.0018 | - | 0.0009987 | ADIPOQ |
| chr3 | rs141205818 | 186572480 | 3q27.3 | A/C | nonsynonymous | 1 | 0.0018 | 1 | 1 | 2 | 0.0018 | - | 0.0006658 | ADIPOQ |
| chr4 | rs61733139 | 100504566 | 4q23 | G/C | nonsynonymous | 1 | 0.053453 | 0 | 1 | 1 | 0.03 | 0.053453 | 0.0013316 | MTTP |
| chr4 | rs2306986 | 100504575 | 4q23 | G/C | nonsynonymous | 0.038395717 | 0.13 | 19 | 12 | 31 | 0.13 | 0.089895 | 0.0652463 | MTTP |
| chr4 | rs61750974 | 100512392 | 4q23 | G/A | nonsynonymous | 0.396039604 | 0.01 | 1 | 0 | 1 | 0.01 | 0.007766 | 0.0039947 | MTTP |
| chr4 | rs17599091 | 100512919 | 4q23 | C/G | nonsynonymous | 0.396039604 | 0.040674 | 1 | 0 | 1 | 0.02 | 0.040674 | 0.0003329 | MTTP |
| chr4 | rs199664737 | 100530116 | 4q23 | G/A | nonsynonymous | 1 | 0.0009 | 0 | 1 | 1 | 0.0009 | - | 0.0003329 | MTTP |
| chr4 | rs2270565 | 141483471 | 4q31.1 | T/A | nonsynonymous | 0.102836596 | 0.07 | 10 | 6 | 16 | 0.07 | 0.059819 | 0.0509321 | UCP1 |
| chr4 | rs45539933 | 141489068 | 4q31.1 | C/T | nonsynonymous | 0.311748758 | 0.09 | 6 | 4 | 10 | 0.09 | 0.072274 | 0.0426099 | UCP1 |
| chr5 | rs7732671 | 149212243 | 5q32 | G/C | nonsynonymous | 0.539428643 | 0.110026 | 6 | 6 | 12 | 0.1 | 0.110026 | 0.0223036 | PPARGC1B |
| chr5 | rs45520937 | 149212430 | 5q32 | G/A | nonsynonymous | 0.828175175 | 0.08 | 10 | 18 | 28 | 0.08 | 0.029543 | 0.036285 | PPARGC1B |
| chr5 | rs17572019 | 149212471 | 5q32 | G/A | nonsynonymous | 0.539428643 | 0.07 | 6 | 6 | 12 | 0.07 | 0.064749 | 0.0173103 | PPARGC1B |
| chr5 | rs11959820 | 149212510 | 5q32 | C/A | nonsynonymous | 1 | 0.09 | 10 | 15 | 25 | 0.09 | 0.082116 | 0.0446072 | PPARGC1B |
| chr5 | rs12659862 | 149213217 | 5q32 | C/T | synonymous | 0.208198528 | 0.0163116 | 2 | 9 | 11 | 0.01 | 0.000538 | 0.0163116 | PPARGC1B |
| chr5 | rs201874416 | 149216003 | 5q32 | G/A | nonsynonymous | 1 | 0.0005 | 0 | 1 | 1 | 0.0005 | - | - | PPARGC1B |
| chr5 | rs45588534 | 149216256 | 5q32 | C/T | synonymous | 0.828175175 | 0.08 | 10 | 18 | 28 | 0.08 | 0.035215 | 0.0426099 | PPARGC1B |
| chr5 | rs45543631 | 149216304 | 5q32 | C/T | synonymous | 0.828175175 | 0.08 | 10 | 18 | 28 | 0.08 | 0.029909 | 0.0412783 | PPARGC1B |
| chr5 | rs139060680 | 149221857 | 5q32 | C/T | synonymous | 0.396039604 | 0.000077 | 1 | 0 | 1 | - | 0.000077 | - | PPARGC1B |
| chr5 | rs144054131 | 149221858 | 5q32 | G/T | nonsynonymous | 0.396039604 | 0.002996 | 1 | 0 | 1 | 0.0023 | 0.000615 | 0.002996 | PPARGC1B |
| chr5 | rs143268818 | 149225335 | 5q32 | C/T | synonymous | 1 | 0.0032 | 1 | 2 | 3 | 0.0032 | 0.000154 | 0.0009987 | PPARGC1B |
| chr8 | rs328 | 19819724 | 8p21.3 | C/G | stopgain | 1 | 0.1 | 7 | 10 | 17 | 0.1 | 0.086345 | 0.0332889 | LPL |
| chr8 | rs193169779 | 145540746 | 8q24.3 | C/T | nonsynonymous | 1 | 0.0005 | 0 | 1 | 1 | 0.0005 | 0.000154 | - | DGAT1 |
| chr8 | rs4731 | 11666337 | 8p23.1 | A/G | nonsynonymous | 1 | 0.065893 | 1 | 1 | 2 | 0.04 | 0.065893 | 0.0053262 | FDFT1 |
| chr8 | rs200217031 | 11667238 | 8p23.1 | C/G | nonsynonymous | 1 | 0.0023302 | 0 | 1 | 1 | - | - | 0.0023302 | FDFT1 |
| chr8 | rs139230476 | 11683543 | 8p23.1 | A/G | nonsynonymous | 1 | 0.0005 | 0 | 1 | 1 | 0.0005 | 0.000077 | 0.0003329 | FDFT1 |
| chr8 | rs79708434 | 11695931 | 8p23.1 | C/G | nonsynonymous | 0.435261349 | 0.0139814 | 4 | 3 | 7 | 0.01 | - | 0.0139814 | FDFT1 |
| chr8 | rs80310078 | 11696115 | 8p23.1 | C/G | nonsynonymous | 0.435261349 | 0.0123169 | 4 | 3 | 7 | 0.01 | - | 0.0123169 | FDFT1 |
| chr10 | rs72559710 | 135342034 | 10q26.3 | G/A | nonsynonymous | 0.523005242 | 0.0033289 | 0 | 2 | 2 | 0.0009 | - | 0.0033289 | CYP2E1 |
| chr11 | rs181017988 | 75480116 | 11q13.5 | A/G | nonsynonymous | 1 | 0.002996 | 0 | 1 | 1 | 0.0014 | - | 0.002996 | DGAT2 |
| chr11 | rs150145390 | 111896242 | 11q23.1 | G/A | nonsynonymous | 0.121080245 | 0.0169774 | 5 | 2 | 7 | 0.01 | 0.000308 | 0.0169774 | DLAT |
| chr11 | rs146700813 | 111910008 | 11q23.1 | C/T | nonsynonymous | 1 | 0.000154 | 0 | 1 | 1 | - | 0.000154 | - | DLAT |
| chr11 | rs150459546 | 68529121 | 11q13.3 | G/A | nonsynonymous | 1 | 0.0013316 | 0 | 1 | 1 | 0.0005 | - | 0.0013316 | CPT1A |
| chr11 | rs139576982 | 821676 | 11p15.5 | G/A | nonsynonymous | 0.396039604 | 0.0009 | 1 | 0 | 1 | 0.0009 | 0.000538 | - | PNPLA2 |
| chr11 | rs200088893 | 823799 | 11p15.5 | C/G | nonsynonymous | 0.28726442 | 0.0026631 | 0 | 3 | 3 | - | 0.000077 | 0.0026631 | PNPLA2 |
| chr11 | rs201573098 | 824813 | 11p15.5 | G/T | nonsynonymous | 0.15919239 | 0.0026631 | 2 | 0 | 2 | - | - | 0.0026631 | PNPLA2 |
| chr11 | rs17848372 | 73716960 | 11q13.4 | C/T | nonsynonymous | 1 | 0.0019973 | 0 | 1 | 1 | 0.0018 | 0.000539 | 0.0019973 | UCP3 |
| chr11 | rs199727434 | 73717970 | 11q13.4 | G/A | nonsynonymous | 1 | 0.0006658 | 0 | 1 | 1 | - | 0.000077 | 0.0006658 | UCP3 |
| chr12 | rs199979885 | 81471954 | 12q21.31 | G/C | nonsynonymous | 1 | 0.0018 | 2 | 3 | 5 | 0.0018 | - | 0.0009987 | ACSS3 |
| chr12 | rs145208016 | 81503421 | 12q21.31 | A/G | nonsynonymous | 0.396039604 | 0.0005 | 1 | 0 | 1 | 0.0005 | - | - | ACSS3 |
| chr12 | rs61745251 | 81528625 | 12q21.31 | C/T | nonsynonymous | 0.52933607 | 0.05336 | 3 | 8 | 11 | 0.05 | 0.05336 | 0.017976 | ACSS3 |
| chr12 | rs17848820 | 109604668 | 12q24.11 | C/T | nonsynonymous | 1 | 0.002996 | 0 | 1 | 1 | 0.0018 | - | 0.002996 | ACACB |
| chr12 | rs184816447 | 109613826 | 12q24.11 | A/G | nonsynonymous | 0.396039604 | 0.0053262 | 1 | 0 | 1 | 0.0018 | - | 0.0053262 | ACACB |
| chr12 | rs17848802 | 109634834 | 12q24.11 | A/G | nonsynonymous | 0.523005242 | 0.03 | 0 | 2 | 2 | 0.03 | 0.022067 | 0.0113182 | ACACB |
| chr12 | rs17848829 | 109678898 | 12q24.11 | C/T | nonsynonymous | 0.396039604 | 0.0023302 | 1 | 0 | 1 | 0.0014 | - | 0.0023302 | ACACB |
| chr12 | rs144673785 | 109684039 | 12q24.11 | G/A | nonsynonymous | 1 | 0.004844 | 0 | 1 | 1 | 0.0027 | 0.004844 | - | ACACB |
| chr12 | rs61752535 | 109684125 | 12q24.11 | G/A | nonsynonymous | 0.396039604 | 0.075581 | 1 | 0 | 1 | 0.05 | 0.075581 | 0.002996 | ACACB |
| chr12 | rs17848835 | 109692053 | 12q24.11 | C/T | nonsynonymous | 0.294321208 | 0.114947 | 0 | 4 | 4 | 0.08 | 0.114947 | 0.0156458 | ACACB |
| chr15 | rs192101674 | 50518191 | 15q21.2 | A/T | nonsynonymous | 0.396039604 | 0.0005 | 1 | 0 | 1 | 0.0005 | - | - | SLC27A2 |
| chr15 | rs6078 | 58833993 | 15q21.3 | G/A | nonsynonymous | 0.404067367 | 0.11 | 27 | 31 | 58 | 0.11 | 0.043646 | 0.1021971 | LIPC |
| chr16 | rs3743788 | 55734106 | 16q12.2 | T/C | nonsynonymous | 0.015142208 | 0.0133156 | 10 | 3 | 13 | 0.01 | 0.000077 | 0.0133156 | SLC6A2 |
| chr16 | rs201308435 | 55736257 | 16q12.2 | G/A | synonymous | 0.396039604 | 0.0036618 | 1 | 0 | 1 | 0.0023 | - | 0.0036618 | SLC6A2 |
| chr17 | rs70937018 | 17723019 | 17p11.2 | G/C | nonsynonymous | 0.561602705 | 0.0018 | 2 | 1 | 3 | 0.0018 | - | 0.0016644 | SREBF1 |
| chr17 | rs200842352 | 80037439 | 17q25.3 | C/A | nonsynonymous | 1 | 0.0049933 | 1 | 2 | 3 | 0.0009 | - | 0.0049933 | FASN |
| chr17 | rs2228306 | 80040034 | 17q25.3 | A/G | nonsynonymous | 1 | 0.03 | 1 | 2 | 3 | 0.03 | 0.022916 | 0.0039947 | FASN |
| chr17 | rs45557233 | 80041950 | 17q25.3 | G/A | nonsynonymous | 0.661266206 | 0.0409454 | 12 | 15 | 27 | 0.03 | 0.000642 | 0.0409454 | FASN |
| chr17 | rs377037839 | 80041953 | 17q25.3 | G/A | nonsynonymous | 0.396039604 | 0.00008 | 1 | 0 | 1 | - | 0.00008 | - | FASN |
| chr17 | rs201182683 | 80045016 | 17q25.3 | T/C | nonsynonymous | 1 | 0.0018 | 0 | 1 | 1 | 0.0018 | - | 0.0009987 | FASN |
| chr17 | rs200374835 | 80048888 | 17q25.3 | C/T | nonsynonymous | 0.396039604 | 0.0005 | 1 | 0 | 1 | 0.0005 | - | - | FASN |
| chr17 | rs141275719 | 80049206 | 17q25.3 | C/T | nonsynonymous | 1 | 0.000232 | 0 | 1 | 1 | - | 0.000232 | - | FASN |
| chr17 | rs77857768 | 17425684 | 17p11.2 | C/T | nonsynonymous | 0.523005242 | 0.0041 | 0 | 2 | 2 | 0.0041 | 0.000231 | 0.0036618 | PEMT |
| chr19 | rs151010811 | 50209247 | 19q13.33 | G/A | nonsynonymous | 0.396039604 | 0.0006658 | 1 | 0 | 1 | - | - | 0.0006658 | CPT1C |
| chr19 | rs201344756 | 42906851 | 19q13.2 | G/A | nonsynonymous | 1 | 0.0005 | 0 | 1 | 1 | 0.0005 | - | - | LIPE |
| chr19 | rs70937099 | 42907064 | 19q13.2 | A/G | nonsynonymous | 1 | 0.01 | 3 | 5 | 8 | 0.01 | 0.000154 | 0.008988 | LIPE |
| chr19 | rs34052647 | 42911769 | 19q13.2 | G/A | nonsynonymous | 0.142816765 | 0.0289614 | 8 | 5 | 13 | 0.02 | 0.001076 | 0.0289614 | LIPE |
| chr19 | rs200004508 | 42911883 | 19q13.2 | C/T | nonsynonymous | 1 | 0.0005 | 0 | 1 | 1 | 0.0005 | - | - | LIPE |
| chr19 | rs201302932 | 42912244 | 19q13.2 | C/T | nonsynonymous | 1 | 0.0005 | 0 | 1 | 1 | 0.0005 | 0.000077 | - | LIPE |
| chr19 | rs201721725 | 42914527 | 19q13.2 | C/T | nonsynonymous | 1 | 0.0005 | 0 | 1 | 1 | 0.0005 | - | - | LIPE |
| chr20 | rs201515743 | 25011400 | 20p11.21 | T/C | nonsynonymous | 0.396039604 | 0.0005 | 1 | 0 | 1 | 0.0005 | - | - | ACSS1 |
| chr20 | rs147336539 | 25004229 | 20p11.21 | C/T | nonsynonymous | 0.523005242 | 0.0076565 | 0 | 2 | 2 | 0.0023 | 0.000231 | 0.0076565 | ACSS1 |
| chr22 | rs201991869 | 42264732 | 22q13.2 | C/T | nonsynonymous | 1 | 0.0005 | 0 | 1 | 1 | 0.0005 | - | - | SREBF2 |
| chr22 | rs377649542 | 42276853 | 22q13.2 | G/A | nonsynonymous | 1 | 0.0003329 | 0 | 1 | 1 | - | - | 0.0003329 | SREBF2 |
| chr22 | rs2228313 | 42293140 | 22q13.2 | G/C | nonsynonymous | 0.584059131 | 0.07 | 7 | 8 | 15 | 0.07 | 0.061895 | 0.01498 | SREBF2 |
| chr22 | rs17848351 | 42294751 | 22q13.2 | G/C | nonsynonymous | 0.15919239 | 0.0023 | 2 | 0 | 2 | 0.0023 | 0.000077 | 0.0013316 | SREBF2 |
| chr22 | rs17848337 | 42300948 | 22q13.2 | C/A | synonymous | 0.76476878 | 0.06 | 6 | 7 | 13 | 0.06 | 0.055102 | 0.0123169 | SREBF2 |
| chr22 | rs370196827 | 42301596 | 22q13.2 | C/T | nonsynonymous | 1 | 0.000077 | 0 | 1 | 1 | - | 0.000077 | - | SREBF2 |
| chr22 | rs1800234 | 46615880 | 22q13.31 | T/C | nonsynonymous | 0.157676337 | 0.0223036 | 6 | 3 | 9 | 0.02 | 0.000615 | 0.0223036 | PPARA |
| chr22 | rs2269383 | 51012775 | 22q13.33 | C/T | nonsynonymous | 1 | 0.06 | 10 | 15 | 25 | 0.06 | 0.033605 | 0.0532623 | CPT1B |
| chr22 | rs2076213 | 44322922 | 22q13.31 | T/G | nonsynonymous | 0.386819543 | 0.08004 | 3 | 2 | 5 | 0.08 | 0.08004 | 0.0276298 | PNPLA3 |
| chr22 | rs2076212 | 44322970 | 22q13.31 | G/T | nonsynonymous | 0.121080245 | 0.142396 | 5 | 2 | 7 | 0.11 | 0.142396 | 0.0289614 | PNPLA3 |
| chr22 | rs143392071 | 44328930 | 22q13.31 | A/G | nonsynonymous | 0.396039604 | 0.0023 | 1 | 0 | 1 | 0.0023 | - | 0.0013316 | PNPLA3 |
| chr22 | rs201343851 | 44332993 | 22q13.31 | G/A | nonsynonymous | 0.396039604 | 0.0003329 | 1 | 0 | 1 | - | - | 0.0003329 | PNPLA3 |
