# Supplementary material for: Association of MTTP gene variants with pediatric NAFLD: A candidate-gene-based analysis of single nucleotide variations in obese children
Source: PLoS One. 2017 Sep 27;12(9):e0185396. doi: 10.1371/journal.pone.0185396 (PMC5617203; doi:10.1371/journal.pone.0185396)
Supplement: S3 Table — The mutations were compared using Fisher's exact test. The 26 SNVs located in 16 genes were enriched in the NAFLD subjects compared to in-house controls (all P < 0.05). (DOC) [file pone.0185396.s003.doc]

**S3 Table.** The distributions of 26 SNVs and the comparison between NAFLD, non-ANFLD groups and inhouse controls (subjects of Han Chinese ethnicity).

| Gene Information | | | | | Mutations | | | Comparison between groups | | | | | | | |
| --- | --- | --- | --- | --- | --- | --- | --- | --- | --- | --- | --- | --- | --- | --- | --- |
| Gene | Pos | Chr | Ref | Alt | NAFLD  (N=39) | non-NAFLD  (N=61) | In-house control  (N=800) | 1000  genome | ESP6500 | iGeneTech  Base | PolyPhen 2 | MutationTaster | GERP++ | Fisher.P | |
|  |  |  |  |  |  |  |  |  |  |  |  |  |  | NAFLD vs  non-NAFLD | NAFLD vs in-house control |
| ACSS3 | rs145208016 | 12q21.31 | A | G | 1 | 0 | 0 | 0.0005 | - | - | 0.993 | 1 | 6.07 | 0.396039604 | 0.047619048 |
| SREBF1 | rs70937018 | 17p11.2 | G | C | 2 | 1 | 0.00625 | 0.0018 | - | 0.0016644 | - | 1 | 1.72 | 0.561602705 | 0.0125468 |
| SREBF2 | rs17848337 | 22q13.2 | C | A | 6 | 7 | 0.05987 | 0.06 | 0.055102 | 0.0123169 | - | 1 | 3.18 | 0.76476878 | 0.0312597 |
| ACACB | rs184816447 | 12q24.11 | A | G | 1 | 0 | 0 | 0.0018 | - | 0.0053262 | 0.005 | 1 | 5.78 | 0.396039604 | 0.047619048 |
| ACACB | rs17848829 | 12q24.11 | C | T | 1 | 0 | 0 | 0.0014 | - | 0.0023302 | 1 | 1 | 4.99 | 0.396039604 | 0.047619048 |
| ACACB | rs61752535 | 12q24.11 | G | A | 1 | 0 | 0 | 0.05 | 0.075581 | 0.002996 | 0.996 | 1 | 4.3 | 0.396039604 | 0.047619048 |
| FASN | rs2228306 | 17q25.3 | A | G | 1 | 2 | 0 | 0.03 | 0.022916 | 0.0039947 | 0.005 | 0.999 | 3.67 | 1 | 0.047619048 |
| FASN | rs377037839 | 17q25.3 | G | A | 1 | 0 | 0 | - | 0.00008 | - | 0.997 | 1 | 2.31 | 0.396039604 | 0.047619048 |
| FASN | rs200374835 | 17q25.3 | C | T | 1 | 0 | 0 | 0.0005 | - | - | 0.992 | 1 | 3.96 | 0.396039604 | 0.047619048 |
| SLC27A2 | rs192101674 | 15q21.2 | A | T | 1 | 0 | 0 | 0.0005 | - | - | 0.001 | 1 | -7.6 | 0.396039604 | 0.047619048 |
| PPARGC1B | rs144054131 | 5q32 | G | T | 1 | 0 | 0 | 0.0023 | 0.000615 | 0.002996 | 0.998 | 0.74 | 2.58 | 0.396039604 | 0.047619048 |
| PPARGC1B | rs143268818 | 5q32 | C | T | 1 | 2 | 0 | 0.0032 | 0.000154 | 0.0009987 | - | 1 | -5.37 | 1 | 0.047619048 |
| ADIPOQ | rs138773406 | 3q27.3 | C | A | 1 | 0 | 0 | 0.0018 | - | 0.0009987 | 0.002 | 1 | -5.24 | 0.396039604 | 0.047619048 |
| ADIPOQ | rs141205818 | 3q27.3 | A | C | 1 | 1 | 0 | 0.0018 | - | 0.0006658 | 0.001 | 0.615 | 3.47 | 1 | 0.047619048 |
| CPT2 | rs142600166 | 1p32.3 | G | A | 1 | 0 | 0 | 0.0005 | 0.000538 | - | 0.001 | 1 | 0.274 | 0.396039604 | 0.047619048 |
| PNPLA3 | rs2076213 | 22q13.31 | T | G | 3 | 2 | 0.005 | 0.08 | 0.08004 | 0.0276298 | 0 | 0 | -2.13 | 0.386819543 | 0.013029896 |
| PNPLA3 | rs2076212 | 22q13.31 | G | T | 5 | 2 | 0.04375 | 0.11 | 0.142396 | 0.0289614 | 0 | 0 | -6.85 | 0.121080245 | 0.0332519 |
| PNPLA3 | rs143392071 | 22q13.31 | A | G | 1 | 0 | 0 | 0.0023 | - | 0.0013316 | 0.994 | 0.993 | 4.54 | 0.396039604 | 0.047619048 |
| PNPLA3 | rs201343851 | 22q13.31 | G | A | 1 | 0 | 0 | - | - | 0.0003329 | 0 | 1 | -9.4 | 0.396039604 | 0.047619048 |
| PNPLA2 | rs139576982 | 11p15.5 | G | A | 1 | 0 | 0 | 0.0009 | 0.000538 | - | 1 | 1 | 4.48 | 0.396039604 | 0.047619048 |
| MTTP | rs61750974 | 4q23 | G | A | 1 | 0 | 0 | 0.01 | 0.007766 | 0.0039947 | 0.98 | 0.951 | 5.07 | 0.396039604 | 0.047619048 |
| MTTP | rs17599091 | 4q23 | C | G | 1 | 0 | 0 | 0.02 | 0.040674 | 0.0003329 | 0.011 | 1 | 3.47 | 0.396039604 | 0.047619048 |
| APOB | rs184512808 | 2p24.1 | T | C | 1 | 0 | 0 | 0.0005 | - | 0.0013316 | 0.999 | 1 | 5.76 | 0.396039604 | 0.047619048 |
| APOB | rs186544754 | 2p24.1 | C | A | 3 | 4 | 0.005 | 0.0014 | - | 0.0016644 | 0.063 | 0.726 | 2.01 | 1 | 0.013029896 |
| SLC6A2 | rs3743788 | 16q12.2 | T | C | 10 | 3 | 0.034 | 0.01 | 0.000077 | 0.0133156 | 0 | 0.977 | 4.01 | 0.015142208 | 0 |
| DLAT | rs150145390 | 11q23.1 | G | A | 5 | 2 | 0.01625 | 0.01 | 0.000308 | 0.0169774 | 0.064 | 1 | -1.44 | 0.121080245 | 0.013861483 |
| TNF | - | 6p21.33 | G | A | 1 | 1 | 0 | - | - | 0.0006658 | 0.001 | 1 | -0.576 | 1 | 0.047619048 |
